# The Action of Pilocarpin upon Horses

**Published:** 1883-01

**Authors:** 


THE ACTION OF PILOCARPIN UPON HORSES.—After a subcutaneous injection of philocarpin ( gr i. ) the saliva begins to flow and continues for about an hour. The injection of a large dose (gr iii.) will cause the secretion of one or two quarts of saliva in a short time. There is also a considerable discharge from the conjunctival and nasal mucous membranes. The temperature rises from two-fifths to one degree F. The heart beats faster by five to ten pulsations. General perspiration does not often occur. Frequent urination will be noticed. After several days' use of pilocarpin the fecis becomes softer. The horse does not seem to be weakened or made less active by the drug. It has been found somewhat useful in diminishing dropsy from kidney or heart disease also in lessening the amount of pleura effusion (in dogs.) Its action on the heart is weakening—a fact to be remembered.

